# Early propranolol treatment induces lung heme-oxygenase-1, attenuates metabolic dysfunction, and improves survival following experimental sepsis

**DOI:** 10.1186/cc12889

**Published:** 2013-09-10

**Authors:** Joel Wilson, David Higgins, Haley Hutting, Natalie Serkova, Christine Baird, Ludmila Khailova, Kelly Queensland, Zung Vu Tran, Lindsay Weitzel, Paul E Wischmeyer

**Affiliations:** 1 Department of Anesthesiology, University of Colorado School of Medicine, Aurora, CO 80045, USA

## Abstract

**Introduction:**

Pharmacological agents that block beta-adrenergic receptors have been associated with improved outcome in burn injury. It has been hypothesized that injuries leading to a hypermetabolic state, such as septic shock, may also benefit from beta-blockade; however, outcome data in experimental models have been contradictory. Thus, we investigated the effect of beta-blockade with propranolol on survival, hemodynamics, lung heat shock protein (HSP) expression, metabolism and inflammatory markers in a rat cecal ligation and puncture (CLP) model of sepsis.

**Methods:**

Sprague-Dawley rats receiving either repeated doses (30 minutes pre-CLP and every 8 hours for 24 hours postoperatively) of propranolol or control (normal saline), underwent CLP and were monitored for survival. Additionally, lung and blood samples were collected at 6 and 24 hours for analysis. Animals also underwent monitoring to evaluate global hemodynamics.

**Results:**

Seven days following CLP, propranolol improved survival versus control (*P *< 0.01). Heart rates in the propranolol-treated rats were approximately 23% lower than control rats (*P *< 0.05) over the first 24 hours, but the mean arterial blood pressure was not different between groups. Metabolic analysis of lung tissue demonstrated an increase in lung ATP/ADP ratio and NAD+ content and a decreased ratio of polyunsaturated fatty acids to monounsaturated fatty acids (PUFA/MUFA). Cytokine analysis of the inflammatory cytokine tumor necrosis factor alpha (TNF-alpha) demonstrated decreased expression of TNF-alpha in both lung and plasma at 24 hours post CLP induced sepsis. Finally, propranolol led to a significant increase in lung hemeoxygenase-1 expression, a key cellular protective heat shock protein (HSP) in the lung. Other lung HSP expression was unchanged.

**Conclusions:**

These results suggest that propranolol treatment may decrease mortality during sepsis potentially via a combination of improving metabolism, suppressing aspects of the inflammatory response and enhancing tissue protection.

## Introduction

Pharmacological agents that block beta-adrenergic receptors are frequently used in critically ill patients. Over 30 years ago Berk *et al*. demonstrated that beta-blockers may reduce mortality from both experimental and clinical sepsis and shock [1]. This hypothesis has been revisited recently with new data demonstrating cardiac and metabolism related-benefits to beta-blocker therapy in experimental and clinical critical care settings. Specifically, beta blockade has been used to prevent catecholamine-mediated hypermetabolism and improve cardiac performance in critically ill patients suffering from severe trauma or burn injury [2-6]. These beneficial effects were not associated with any increase in the incidence of hypotension, infection or inflammation [5,7].

Recent reviews propose that beta-blockers may have protective effects in septic patients [8-10]. Although clinical data are mainly limited to case reports, a recent study demonstrated that patients receiving beta-blockers who subsequently develop sepsis experienced a survival advantage compared to patients not previously receiving beta blocker therapy [11]. However, laboratory data indicate conflicting results on the role of beta-blockers in improving survival from sepsis. While it has been hypothesized that the potential beneficial effects of beta-blocker therapy in sepsis are in part due to immune regulation, the effect of beta-blocker therapy on cytokine expression is unclear. Conflicting results have shown that beta-blockade can lead to either an increase or decrease in cytokine expression and immune regulation depending on the experimental model, class of beta blockade (specific versus non-specific) and where the cytokine was measured (organ, plasma and so on) [8]. Confounding all of the pre-clinical data is the fact that these studies utilize a wide range of sepsis models, doses, co-interventions (such as catecholamines), beta-blocker classes and timing of therapy.

Although the demonstrated survival benefit of beta-blockade during sepsis may be due to direct myocardial protective effects, hemodynamic/catecholamine-mediated changes, or immune regulation, there remain other potential benefits of beta-blockade that have not been explored. One of those effects may involve activation of stress-inducible protein systems, or heat shock proteins (HSPs) that cells use to maintain cellular homeostasis during stress and inflammation. Data found by Herndon *et al *via gene-array for RNA expression indicate that propranolol can upregulate gene-expression for key stress-response proteins in muscle biopsies from burned children [12]. These data showed that a member of the HSP 70 family (GRP70) was significantly upregulated in the muscle of patients treated with propranolol. No further exploration of this potential organ protective pathway examining other stress proteins or other tissues has been described.

Much of the recent experimental data on beta-blockers in sepsis have focused on the myocardial and whole body metabolic effects of propranolol in sepsis and injury [13]. Given the conflicting data on the effects of beta blockade during sepsis on metabolism, survival, cardiac performance and immune regulation, further studies, particularly studies examining other organs such as the lung, are needed.

The aim of this study was to investigate the effect of non-specific beta blockade with propranolol on cecal ligation and puncture (CLP)-induced sepsis in the rat and to examine the effect of this therapy on survival, overall hemodynamics and, specifically, on lung tissue cytokine expression, lung metabolism and lung HSP expression.

## Materials and methods

### Animals and experimental protocol

The animal experiments described in this paper were performed in adherence to the National Institute of Health guidelines for the use of experimental animals. The Animal Care and Use Committee of the University of Colorado, Denver, approved all animal protocols. All experiments were also conducted and the animals cared for in accordance with the Guiding Principles for Research Involving Animals and Human Beings of the American Physiological Society.

Animals used in these experiments were male Sprague-Dawley rats with a body weight between 300 and 350 g purchased from Harlan (Madison, WI, USA). They were maintained on a standard diet (Teklad rat chow; Harlan) and water *ad libitum*. They were allowed an acclimatization period of at least 14 days before the procedure. They were housed at a constant temperature (69 to 71°F) with light and dark cycles of 10 hours and 14 hours, respectively.

The CLP method was utilized as previously described [14]. Thirty minutes prior to CLP, the animals were divided into two groups and given propranolol (10 mg/kg) through an intraperitoneal injection or normal saline of the same volume and same administration route. Then, following anesthesia with an intraperitoneal injection of ketamine (80 mg/kg) and xylazine (12 mg/kg), a 2 cm incision was made near the lower midline of the abdomen and the cecum was exposed. 30% of the cecum was then ligated using 3.0 silk just below the ileocecal valve (to avoid bowel obstruction). This percentage of cecum ligation was chosen for its reproducible rate of mortality in control animals [15]. The ligated portion of the cecum was then punctured twice with an 18 g needle and a small amount of the contents were expelled into the peritoneal cavity. Following this, the cecum was returned to its original location within the abdomen, and both the muscle and skin layers were closed. No antibiotics were administered; however, 20 cc/kg normal saline was given subcutaneously as fluid resuscitation. The animals were then returned to their cages and allowed access to chow and water *ad libitum*.

### Propranolol administration

Thirty minutes prior to CLP, the animals were randomly divided into two groups and given either propranolol (10 mg/kg) through an intraperitoneal injection or normal saline (non-propranolol treated septic control) of the same volume and same administration route. The doses mentioned above of either propranolol or saline were given every eight hours postoperatively for 24 hours.

### Survival studies

33 animals (16 propranolol-treated and 17 non-propranolol treated septic control animals) were observed at regular intervals for occurrence of mortality over the subsequent seven days and survival time was recorded. Moribund animals (defined as bradycardia to a heart rate less then 40; severe lethargy; and unresponsive to painful stimulation) were sacrificed with a lethal dose of ketamine/xylazine as defined by the University of Colorado Animal Care Committee.

### Hemodynamic monitoring

A second group (n = 4/group) of animals underwent the same procedures and heart rate and blood pressure were recorded at regular intervals to evaluate the hemodynamic effects of propranolol in our CLP model. An invasive hemodynamic monitoring system (HP 78352C monitor, Hewlett Packard, Avondale, PA, USA) via a carotid arterial catheter was utilized.

### Quantification of lung tissue metabolites

A third set of animals (n = 5/group) followed the aforementioned treatments and lung tissue was harvested at 6 hours and 24 hours for analysis of lung tissue metabolism. Snap frozen lung tissue was extracted with perchloric acid as described in detail by Serkova *et al*. [16]. After centrifugation, the supernatants were analyzed by multinuclear NMR metabolomics or magnetic resonance spectroscopy (MRS) as described previously [16]. Tissue-specific metabolites quantified by multinuclear NMR metabolomics included lung tissue ATP/ADP ratio, high energy phosphates (ATP, ADP, nicotinamide adenine dinucleotide (NAD^+^)) and lactate, glutathione and lipids/phospholipids.

### TNF-α analysis

Additional animals (n = 4 per group/time point) were treated via the same procedure as mentioned above; however, they were sacrificed for blood and lung tissue at 6 and 24 hours post CLP. All tissues were washed in 1X PBS after removal and then frozen at -80°C. The plasma and lung tissue was analyzed by ELISA for TNF-α using a Rat TNF-α ELISA kit. (Thermo Scientific Pierce, Rockford, IL, USA)

### Heat shock protein analysis

Lung tissue was also collected at 6 and 24 hours post CLP (n = 4 per group/time point) for HSP expression. All tissues were washed in 1X PBS after removal and then frozen at -80°C. For whole cell protein expression levels, tissues were homogenized with Mammalian Protein Extraction Reagent (M-PER) (Thermo Scientific, Waltham, MA, USA) plus protease inhibitors. Protein was determined and western blots were run to evaluate beta-actin, HSP25, HSP70 and HO-1 protein expression as previously described [17].

### Statistical analysis

As this was a pilot study of the effect of propranolol on lung protection and inflammatory/metabolic response in sepsis, limited preliminary data were available by which to predict an effect on survival in our model. Thus, to attempt to identify a meaningful survival effect we set a difference of 40% in mortality as our baseline desired effect. We performed the sample size calculation using the following formula: n = (2/d2) × Cp, power, where n is the number of mice needed in each group, d is the standard difference (d = target difference/standard deviation) and Cp, power, is a constant defined by a *P*-value of 0.05 and power of 80%. The number of mice required to detect a 40% difference in mortality assuming a standard deviation of 0.4 to 0.5 was 16 to 25. When 16 and 17 animals per group, respectively, were reached analysis of the observed mortality difference was performed and a statistical difference was observed. To minimize animal exposure to this potentially lethal model we chose not to conduct further survival data when a statistically significant mortality difference was achieved. All statistics were run using Microsoft Office Excel (2007) and SAS 9.2. Student's t-tests were performed to determine differences between the means of TNF-α, ATP/ADP ratio, NAD+ content and PUFA/MUFA ratio. Mann Whitney tests were performed to determine differences between heart rate and mean arterial pressure (MAP) variables at specific time points. Averages are reported as mean +/- SD. A Kaplan-Meier analysis and log-rank test were used to analyze survival data. Differences were considered significant if the *P*-value was less than or equal to 0.05.

## Results

### Propranolol treatment improves overall survival without significant change in blood pressures in a rat model of sepsis

**Figure 1:**
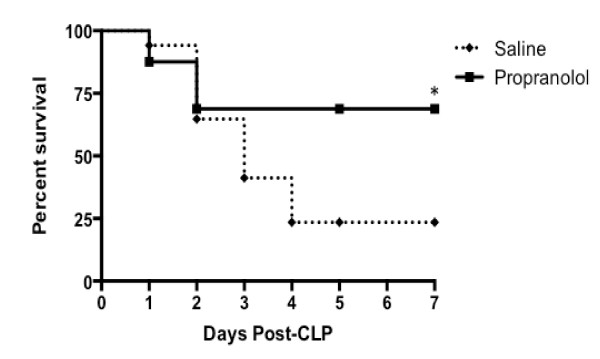
**Kaplan-Meier curve of survival times in control and propranolol groups**. There was significantly increased survival in rats that received propranolol compared with the control group following CLP induced sepsis (log-rank; *P *< 0.05). CLP, cecal ligation and puncture.

**Figure 2:**
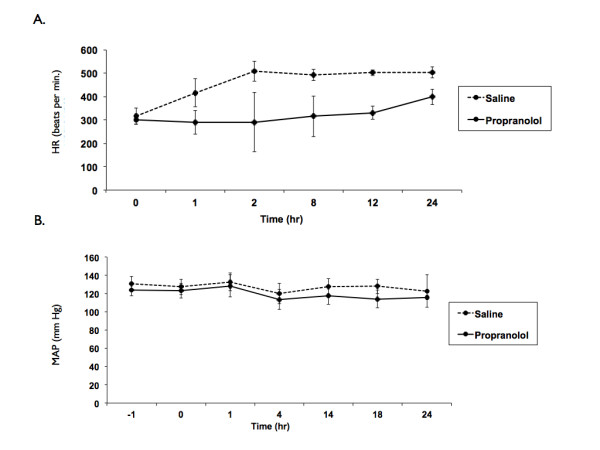
**Heart rate and MAP in control and propranolol groups**. **A**) There was a significant decrease in mean heart rate observed in the propranolol group (n = 4/group, *-*P *< 0.05, **-*P *< 0.01) at 8, 12 and 24 hours. **B**) There was no significant difference in MAP between the groups at any time point (n = 4/group). MAP, mean arterial pressure.

Survival was increased in Sprague-Dawley rats that received propranolol compared with the non-propranolol treated septic control group following CLP-induced sepsis (log-rank test; *P *< 0.05) (Figure 1). Overall mortality at seven days was significantly lower in rats that received propranolol compared to rats that received saline (69% survival rate in propranolol rats (11 out of 16) versus 23% survival in control rats (4 out of 17), *P *< 0.01). Heart rates (Figure 2A) for propranolol-treated rats ranged between 23% to 34% lower than control rats and were significantly lower than control rats at 6, 12 and 24 hours post sepsis onset (*P *< 0.05). However, the MAP remained stable with no statistical difference between the two groups (Figure 2B).

### Propranolol improves metabolic function in lung tissue following sepsis

In order to assess the effect of propranolol on tissue metabolism, NMR metabolism studies were performed in lung tissue at 6 and 24 hours post onset of sepsis (Table 1). No significant changes were observed in lactate content, total glutathione or other fatty acid content (not shown). A decrease in ATP/ADP ratio was observed in both groups versus our previously published non-septic, healthy control animal data (normal untreated control ATP/ADP ratio 3.51 +/- 0.58) [18]. There was a significant increase in lung ATP/ADP ratio in propranolol treated rats at six hours post onset of sepsis compared to untreated septic control rats (*P *< 0.05, 95% CI -0.67 to -0.05) but no significant difference was observed at 24 hours. There was significantly increased lung NAD+ content in propranolol treated rats at 24 hours post onset of sepsis compared to the untreated septic control rats (*P *< 0.01, 95% CI -0.59 to -0.07). Also, there was a significant increase in the lung [PUFA/MUFA] ratio in propranolol-treated rats at 24 hours post onset of sepsis (*P *< 0.05, 95% CI -2.2 to -0.06).

**Table 1:** Effect of propranolol on metabolic function in the lung following sepsis

Metabolite	Saline 6h	Propranolol 6h	Saline 24h	Propranolol 24h
ATP/ADP Ratio	1.35 +/- 0.09	1.63 +/- 0.29^a^	2.03 +/- 0.47	1.67 +/- 0.24
NAD+	0.78 +/- 0.13	0.64 +/- 0.15	0.88 +/- 0.11	1.2 +/- 0.23^b^
PUFA/MUFA	4.14 +/- 1.69	5.83 +/- 2.75	2.42 +/- 0.82	3.53 +/- 0.56^b^

All values are reported as mean +/- S.D. and expressed as micromoles/gram of tissue except for ratio (ATP/ADP). ^a^*P *< 0.05 when compared to saline at 6 hr. ^b^*P *< 0.05 when compared to saline at 24 hour. (n = 5/group/timepoint). NAD^+^, nicotinamide adenine dinucleotide; PUFA/MUFA, polyunsaturated fatty acids to monounsaturated fatty acids.

### Propranolol reduces TNF-α expression in lung and plasma

**Figure 3:**
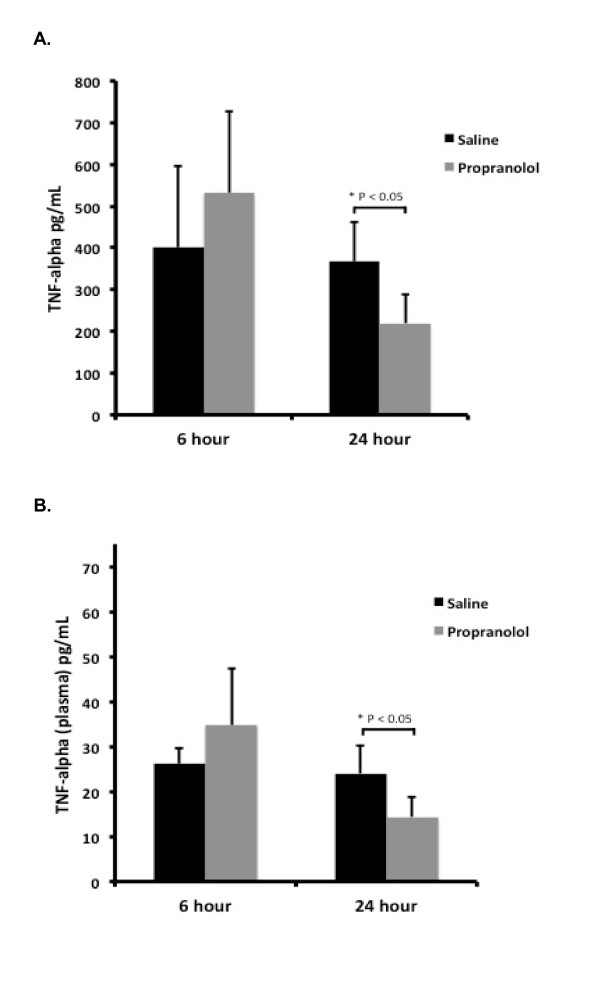
**Effect of propranolol on TNF-α expression**. **A) **Lung TNF-α in control and propranolol groups. There were similar levels of TNF-α between groups at 6 hours. At 24 hours post sepsis onset propranolol treated rats had significantly lower TNF-α compared to saline rats at 24 hours. **B**) Plasma TNF-α in control and propranolol groups. At 24 hours post sepsis onset propranolol treated rats had significantly lower TNF-α compared to saline treated rats at 24 hours. Error bars represent standard deviation and significance markers shown by brackets on figure (n = 4/group).

TNF-α was analyzed to assess the effect of propranolol on pulmonary and systemic inflammation. ELISA of lung homogenates revealed similar levels of TNF-α between groups at six hours but decreased levels of TNF-α at 24 hours post sepsis onset in propranolol treated rats compared to control rats (Figure 3A, P <0.05, 95% CI -293.4 to -1.7). Also, there was a significant decrease in lung TNF-α concentration in propranolol treated rats at 24 hours when compared to six hours post sepsis onset (P <0.05, 95% CI -19.1 to -0.2). The untreated septic control rats did not demonstrate a significant decrease from 6 to 24 hours. Plasma TNF-α levels were also decreased in propranolol treated rats when compared with untreated septic control rats (Figure 3B, P <0.05) at 24 hours post sepsis onset.

### Propranolol increases HO-1 expression, but not other heat shock protein expression in the lung following sepsis

**Figure 4:**
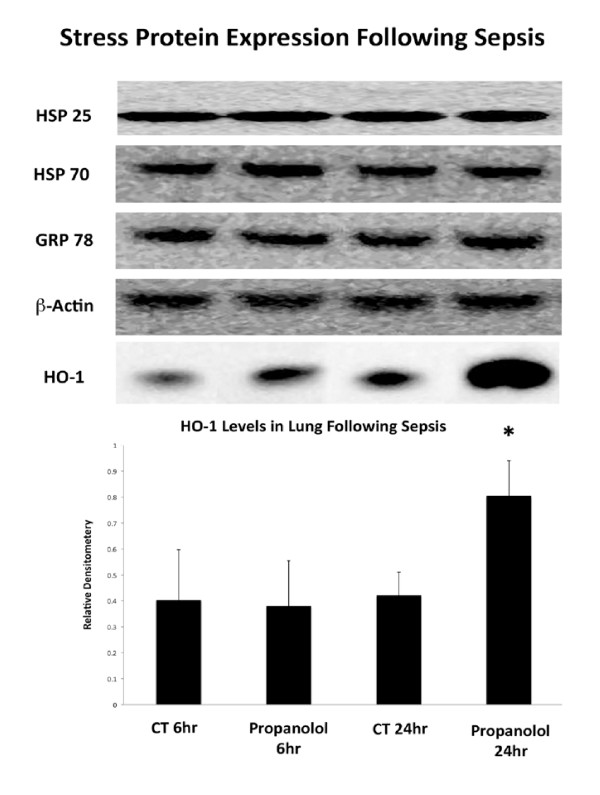
**Heat shock protein (HSP) expression in propranolol and control groups**. Mean relative densitometry of all HSPs normalized to beta-actin. No changes in HSP expression were noted, with the exception of HO-1 expression. There were similar levels of HO-1 between groups at 6 hours. At 24 hours post sepsis onset propranolol treated rats had significantly higher levels of HO-1 compared to saline treated rats (*-*P *< 0.05). Error bars represent standard deviation. Image representative of HSP expression and beta-actin from the lungs in saline and propranolol groups at 6 and 24 hours. Error bars represent standard deviation (n = 4/group).

Levels of HSPs were analyzed in the lung at 6 and 24 hours post sepsis onset. This analysis included HSP70, HSP 25, GRP 78 and HO-1. These specific proteins were chosen because they have been shown to be key in lung protection in our previous experimental sepsis data (HSP 70, HSP 27, HO-1) [18,19] and propranolol has been shown to induce these proteins in the muscle of burned pediatric patients (HSP 70, GRP 78) [12]. As shown in Figure 4, propranolol treatment did not affect lung HSP expression, with the exception of HO-1 (hemeoxygenase-1 or HSP 32). Similar levels of HO-1 and all other measured HSPs were observed between the groups at six hours. However, at 24 hours following sepsis onset, propranolol treated rats had significantly increased levels of lung HO-1 compared to untreated septic control rats (Figure 4, *P *< 0.05, 95% CI -0.8 to -0.02).

## Discussion

This study demonstrates that non-specific beta-blockade with propranolol improves survival in septic rats without evidence of hemodynamic compromise. Furthermore, for the first time we show that propranolol may improve metabolic function in the lungs with significant increases in the ATP/ADP ratio and NAD+ content and a significant decrease in the PUFA/MUFA ratio. Additionally, propranolol affected cytokine expression by decreasing TNF-α levels in both plasma and lung samples. Also, this study is the first to show that propranolol can increase the key tissue protective protein HO-1 in the lung following sepsis.

Our data are the first to show that non-specific beta blockade with propranolol can prolong survival in an experimental sepsis model. While few published studies of beta-blockade in experimental sepsis report mortality data, the reports are conflicting. This is likely due to variability in animal sepsis models, specificity of beta-receptor blockade, dosage and timing of treatment [20]. In a CLP rat model of sepsis similar to our study, beta-1 specific blockade demonstrated a clear survival advantage [21]. Conversely, in a CLP murine model of sepsis where non-specific beta blockade was used, mortality was increased in propranolol treated mice [22]. Unlike our study, however, these studies in mice did not utilize any type of hemodynamic monitoring and were unable to assess if there were propranolol-induced changes in local or systemic perfusion. Additionally, in these studies beta-blockade was initiated at various time points often at the time of surgery or following the procedure. In our study, which demonstrated improved survival, propranolol treatment was initiated prior to the CLP procedure which is consistent with a recent study by Ackland *et al*. demonstrating that beta-blockade before but not after induction of sepsis provided a significant survival advantage [21]. Clinical studies on the survival benefit of beta-blockade in sepsis are limited to only a few case reports and a recent study demonstrating that patients previously on chronic beta-blocker therapy had decreased mortality when admitted to the ICU for sepsis compared to patients not on chronic beta-blockers. Future clinical studies will hopefully shed light on this issue. Currently, there is at least one clinical trial ongoing to investigate outcomes of beta-blockade in sepsis [23].

One of the major limitations of non-specific beta-blockade is a potential effect on local and systemic perfusion and in the clinical setting ICU physicians are constantly monitoring hemodynamics and adjusting treatment accordingly [13]. For this reason, this study was carried out with continuous cardiac monitoring. Although heart rate was decreased by propranolol treatment in our data, MAP remained stable and did not differ between groups. This is in line with other similar studies in the rat CLP model where significant reduction in heart rates by beta blockade was not associated with hypotension or decreased cardiac output [24,25]. It has been proposed that beta-blockers improve survival in part by decreasing heart rate and improving stroke volume, thereby safely reducing cardiac work but this has yet to be confirmed in a multicenter trial. A further limitation is that the first dose of beta-blocker was given 30 minutes prior to the onset of CLP. Thus, this very proximal pre-treatment dose may not be a realistic replication of some forms of sepsis in the clinical setting, when the time of onset of sepsis can often not be predicted. A more ideal comparison of our model would be in the major surgery, burn or trauma patient where sepsis commonly occurs following the initial insult and proximal pre-treatment (that is, pre-operatively) or long term therapy (such as is commonly used in burn injury) may allow for the beta-blocker to be present prior to sepsis initiation. Further experiments examining post-treatment with propranolol are warranted.

Sepsis is accompanied by metabolic changes resulting in a catabolic state of increased energy expenditure, hyperglycemia and muscle loss, which can be modulated via beta-receptors. This study demonstrated that in a rat model, beta-blockade may improve the metabolic and physiological alterations caused during sepsis by increasing the ATP/ADP ratio in the lungs. Additionally, in this study lung NAD+, a marker of enhanced mitochondrial oxidative phosphorylation [16], was increased at 24 hours post-CLP in the propranolol-treated rats. This energy from glucose and fatty acid metabolism is transferred to NAD^+ ^by reduction to NADH, as part of beta oxidation, glycolysis and the citric acid cycle. The electrons carried by the NADH that is produced in the cytoplasm are transferred into the mitochondrion (to reduce mitochondrial NAD^+^) by mitochondrial shuttles, such as the malate-aspartate shuttle. The mitochondrial NADH is then oxidized in turn to NAD^+ ^by the electron transport chain, which pumps protons across a membrane and generates ATP through oxidative phosphorylation. As such, for sepsis, increased NAD+ concentrations show the shift towards ATP production and shift to oxidative phsopshorylation (as confirmed by increased [ATP/ADP] ratios). It is unclear why the differences in NAD+ are not evident at six hours post-sepsis in the two groups, but become more pronounced at 24 hours post-sepsis injury. Similarly, the ATP/ADP ratio is siginificantly different at six hours, but these differences are not observed at 24 hours. Additional targeted metabolic research will be required to understand further the role of propranolol on metabolic function in the lung following sepsis. This may also include the need for later timepoints following the septic insult to understand the kinetics of these metabolic changes further. Another marker that was altered in propranolol-treated rat lungs was the PUFA/MUFA ratio. PUFA/MUFA ratio is a surrogate marker of lipid peroxidation [16,26]. This result indicates that propranolol administration is having an anti(per)oxidant effect on the rats.

During sepsis, unregulated inflammatory cytokines, including the inflammatory cytokine TNF-α, can result in organ failure and severe pathologic inflammation [9]. This study demonstrated that non-selective beta-blockade decreased TNF-α levels both in plasma and in lung tissue at 24 hours post-induction of sepsis. Studies exist showing both an increase and a decrease in TNF-α levels following propranolol treatment. Recent studies in the CLP rat model and beta-1 blockade showed a decrease in TNF-α levels measured in plasma and intraperitoneal fluid [24,27]. In other studies of experimental sepsis, TNF-α was either unchanged [25] or increased [28]. These discrepancies in TNF-α expression are most likely due to the location and timing of cytokine measurement or the type of beta-receptor blocked. Sepsis has been described as displaying a variety of responses within the body compartments [29] and different microenvironments of the body are likely to see a relative increase or decrease in inflammatory cytokines due to sepsis [13]. Further, significant temporal changes in cytokine expression were observed in a rat CLP model of sepsis and timing of cytokine sampling must be considered [27]. Also, TNF-α expression may be different with a non-specific beta-blocker as used in this study when compared with studies utilizing beta-1 specific blockade. The mechanisms of beta-adrenergic immune modulation are not well understood but are thought to occur through regulation of NF-κB expression [9,27]. Studies suggest that immune modulation can be either pro-inflammatory or anti-inflammatory based on whether beta2 or beta1 receptors are blocked, respectively, [9] and the results of our study warrant future studies of this complex immune modulation.

This study is the first to describe increased HO-1 levels in the lungs of septic rats treated with a beta-blocker. Although not all studies show a beneficial role for HO-1 overexpression [30,31], the vast majority of the literature demonstrates that it confers profound protection in several models of lung injury, as well as systemic inflammatory diseases, such as sepsis [32]. HO-1 has been shown to have cytoprotective effects through regulation of apoptosis as well as anti-inflammatory effects, such as decreasing the levels of TNF-α in serum [33]. The potential use of HO-1 clinically has been suggested using gene delivery methods, but the ability to up-regulate HO-1 expression using a beta-blocker would be a promising alternative to difficult gene-therapy delivery methods and warrants further investigation.

## Conclusions

In conclusion, this study demonstrated a significant survival advantage for septic rats given a non-specific beta-blocker. This outcome benefit may be due, not only to previously described effects on the myocardium and muscle, but also benefits on lung metabolism, immune regulation and enhanced pulmonary HSP expression. Further studies are needed to determine the mechanisms, dosages and timing of beta-blockade that may optimally improve outcome in sepsis. This study suggests that propranolol treatment could be beneficial to decrease mortality from sepsis in humans.

## Key messages

• Early treatment (30 minutes pre-injury and dosed for the first 24 hours) with propranolol can improve survival in rodents following polymicrobial sepsis.

• Propranolol administration during septic shock reduced heart rate 20% to 30% without a significant effect on blood pressure.

• Propranolol administration during septic shock can reduce expression of TNF-α in lung and plasma.

• Propranolol administration during septic shock can attenuate lung tissue metabolic dysfunction.

• Propranolol administration during septic shock can induce protective HO-1 (HSP 32) expression.

## Abbreviations

CLP: cecal ligation and puncture; ELISA: enzyme-linked immunosorbent assay; HO-1: hemeoxygenase-1; HSP: heat shock protein; MAP: mean arterial pressure; MRS: magnetic resonance spectroscopy; NAD^+^: nicotinamide adenine dinucleotide; NF-κB: nuclear factor-kappa beta; NMR: nuclear magnetic resonance; PBS: phosphate-buffered saline; PUFA/MUFA: polyunsaturated fatty acids to monounsaturated fatty acids; TNF-α: tumor necrosis factor-alpha.

## Competing interests

The authors declare that they have no competing interests.

## Authors' contributions

JW assisted with the conception of the experimental design, performed animal survival studies, cytokine analysis and heat shock protein analysis. DH assisted with the conception of the experimental design, performed heat shock protein analysis and did significant writing and statistical analysis of the manuscript. HH assisted with the conception of the experimental design, performed all hemodynamic measures experiments and assisted in survival studies. NS performed all NMR based metabolomics analysis. CB performed heat shock protein analysis, cytokine analysis, sample preparation for metabolic analysis, and writing of the manuscript. LK performed cytokine analysis and assisted with heat shock protein analysis. KQ assisted with animal procedures, maintenance of animal protocols, and sample/tissue collection and processing. ZVT was the Senior Biostatistician and provided extensive assistance with statistical analysis and interpretation of data. LW assisted with data synthesis and analysis. PW conceived the initial concept for the experimental design and research question and oversaw and directed the experimental design and contributed significantly to the writing of the manuscript. All authors have read and approved the final manuscript.
